# Long-Term Functional Outcomes and Quality of Life After Microvascular Reconstruction of Ankle and Foot Defects: A Monocentric Controlled Cohort Study

**DOI:** 10.3390/life15050775

**Published:** 2025-05-13

**Authors:** Sarah Pfeifenberger, Andrzej Hecker, Nikolaus Watzinger, Maximilian Moshammer, Anna-Lisa Pignet, Alexander Draschl, Ron Martin, Charalambos Louca, Lars-Peter Kamolz, Stephan Spendel

**Affiliations:** 1 Division of Plastic, Aesthetic and Reconstructive Surgery, Department of Surgery, Medical University of Graz, 8036 Graz, Austria; sarah.pfeifenberger@stud.medunigraz.at (S.P.); lars.kamolz@medunigraz.at (L.-P.K.); stephan.spendel@medunigraz.at (S.S.); 2 COREMED—Centre for Regenerative Medicine and Precision Medicine, Neue Stiftingtalstrasse 2, 8010 Graz, Austria; 3 Department of Plastic and Hand Surgery, Burn Care Unit, BG Klinik Bergmannstrost Halle, 06112 Halle, Germany; 4 Department of Plastic and Reconstructive Surgery, Marienhospital Stuttgart, Teaching Hospital of the Eberhard Karls University, 70199 Stuttgart, Germany

**Keywords:** microvascular reconstruction, foot defect, PROM, long-term outcome, health-related quality of life, functionality

## Abstract

Background: Defects of the foot and ankle area pose a significant challenge for both patients and surgeons. While the primary objective of microvascular free flap reconstructions of lower leg defects is limb preservation, there should be an effort to obtain the best functional and aesthetic results possible and to restore the patient’s quality of life. The aim of this study was to investigate the long-term post-operative functional outcome, health-related quality of life, scar quality, and aesthetic satisfaction in patients following microsurgical reconstructions of defects of the foot and ankle area. Methods: We conducted a monocentric, controlled cohort study of adult patients who underwent microsurgical reconstructions for defects of the foot and ankle area between 2006 and August 2022 at our department. As a control, we recruited healthy individuals. Patient-Reported Outcome Measures questionnaires were used to assess long-term results regarding functionality (LEFS: Lower Extremity Functional Scale), health-related quality of life (SF-36: Short-Form 36 Health Survey Questionnaire), scar quality (PSAS: Patient Scar Assessment Scale), aesthetic satisfaction (Aesthetic Likert Scale), pain at rest and activity (10-point Numeric Rating Scale) and usage of footwear. Results: Of the 55 potential patients who had received microvascular free flaps for reconstructions of ankle and foot defects and were eligible for study inclusion, 13 (23.6%) agreed to participate in this study. The study cohort consisted of 84.6% male subjects and the mean follow-up period was 8.6 years (±5.2). Significant moderate limitations were observed with regard to physical function of the lower extremity (LEFS: 42.5 ± 20.5, *p* = 0.002). Significant limitations were found in the SF-36 subscales of pain (55.8 ± 34.5, *p* = 0.019), physical functioning (55.0 ± 29.7, *p* = 0.013), and physical role functioning (38.5 ± 44.0, *p* = 0.006). The study yielded favorable outcomes with regard to aesthetic satisfaction (14.3 ± 4.4) and scar quality (23.5 ± 13.5). Out of all the patients, 61% were required to wear orthopedic shoes or insoles. Conclusions: Patients undergoing microsurgical reconstructions for ankle and foot defects experience moderate long-term physical limitations and persistent pain during activity. Furthermore, we observed a significant long-term impact on specific physical domains of health-related quality of life, whereas mental health seems less affected.

## 1. Introduction

The foot is a specialized part of the body that is primarily responsible for facilitating upright movement. A variety of anatomic structures engage in specific interactions that facilitate the mobility that is a familiar aspect of human movement [1,2]. Defects of the foot disrupt this interaction, thereby imposing a significant limitation on affected patients and rendering treatment challenging. In the context of extensive soft tissue defects, skin grafts are unable to provide their own vascular supply and are, therefore, unsuitable for use in weight-bearing areas of the foot [3]. Local or pedicled flaps can often be an effective solution, but they may not be available in cases of larger or more distally located defects [4]. In such instances, microvascular free flaps may be employed, as they are not only ideal for extensive defects but also allow for the restoration of lost function and provide healthy tissue for a better aesthetic appearance [5,6]. In the event of severe trauma to the lower leg, operating surgeons are frequently confronted with a complex reconstruction challenge. Due to its characteristics, the foot and ankle region is a surgically challenging anatomical structure [7,8]. While defects of the weight-bearing plantar region necessitate robust tissue to withstand high pressure and shear forces, defects of the ankle and the dorsal region require pliable and thinner tissue to adapt to the natural contours of the area [9]. In addition, the foot contains numerous vulnerable structures, including tendons, nerves and blood vessels. The lack of protective tissue, particularly in the dorsal area, renders those structures susceptible to injury and further complicates the reconstruction process [1]. In situations where local reconstructive techniques prove inadequate for safeguarding vital structures, microsurgical reconstruction is necessary [10,11].

The main goal of microvascular free tissue transfers in lower leg defects is the preservation of the limb [11]. Studies found that individuals who underwent reconstructive surgery following foot trauma exhibited a reduced prevalence of physical and mental limitations when compared to those who underwent amputation of the injured foot [12]. However, the goals of lower extremity reconstruction are multifaceted and have enduring effects on the patient’s psychological and functional well-being. It is, therefore, imperative to strive for optimal functional and aesthetic outcomes, in addition to restoring the patient’s quality of life following microvascular reconstruction [13]. In the context of lower extremity reconstruction, the most prevalent microsurgical reconstruction techniques are either muscle or fasciocutaneous flaps. Both types of free tissue transfer have comparable success rates in terms of flap survival, limb salvage, and functional recovery in lower extremities following traumatic injury. However, with regard to aesthetic outcomes, studies indicate that fasciocutaneous flaps yield superior results compared to muscle flaps [14,15,16]. Although no differences were observed in flap contour, bulkiness, color matching, or scarring, patients who received fasciocutaneous flaps for lower extremity reconstructions expressed higher satisfaction with flap texture than those who received muscle flaps. Unlike fasciocutaneous, which typically offer a more natural appearance, muscle flaps require skin grafts that often result in visible textural differences [11,17]. Nevertheless, patients frequently expressed dissatisfaction with the bulkiness and missing contours of the utilized flap following lower extremity reconstruction [17,18]. The aesthetic component is of great importance to patients, as demonstrated by the frequent desire for secondary refinement following lower extremity free flap procedures [19]. This subjective perception of the aesthetic aspect is of particular importance, as patients who reported higher levels of aesthetic satisfaction tend to exhibit superior health-related quality-of-life outcomes [20].

In examining the health-related quality of life, it is essential to consider it within the context of the bio-psycho-social model [21]. This model considers the physical factors that contribute to overall well-being while emphasizing the importance of mental health and social factors [21,22]. A number of studies have demonstrated a strong correlation between enhanced physical capabilities and improved mental health, as well as a strong positive correlation between foot health and quality of life in patients who have suffered lower limb trauma [12,23]. Patients who have experienced severe trauma frequently encounter not only physical limitations and significant pain that impair their functioning and social activities but also mental health conditions such as anxiety and depression [7,24]. Social isolation, inability to work and the stigma associated with disability within society can contribute to these mental health issues [12]. Studies found that patients often experience a significant level of pain following lower limb reconstruction, which serves to exacerbate existing physical limitations [7].

While severe lower extremity defects can have a profound impact on patients’ lives, understanding their disease-specific perspectives and the effects of treatments is crucial for informed decision-making [13]. The aim of this study was to examine the long-term functional outcomes and quality of life in patients who had undergone microvascular reconstructions of the lower extremity, as well as the impact of various factors, including scar quality, pain, and aesthetic satisfaction, from a patient-centric perspective. Long-term data on microvascular reconstruction of the foot and ankle, particularly regarding functionality and quality of life from a patient-centric perspective, remain scarce. The findings of this study provide a comprehensive understanding of the lived experience of patients who have undergone microvascular reconstructions of the foot and ankle region.

## 2. Materials and Methods

This monocentric, controlled cohort study was conducted in accordance with the principles set forth in the Declaration of Helsinki. Approval was granted by the institutional ethics committee (34–446 ex 21/22, 5 August 2022) of the Medical University of Graz. The methods were carried out in accordance with the relevant guidelines and regulations. Informed consent was obtained from all individual participants included in the study.

### 2.1. Patients and Data Collection

We searched the electronic patient records in the institutional database using codes of German Procedure Classification (5–905.0, 5–905.0f, 5–905.0g, 5–925, 5–925.4, 5–925.4f, 5–925.4g, 5–906.0, 5–906.0f, 5–906.0g) to identify patients who had undergone microvascular free flap reconstructions of the foot and ankle region and treated at the Division of Plastic, Aesthetic, and Reconstructive Surgery, Department for Surgery, University Hospital Graz, between January 2006 and August 2022. Subsequently, data were manually retrieved and reviewed from medical and operative reports, outpatient notes, and pre-operative anesthetic records.

The patient-related variables included demographic data (age, gender, body mass index (BMI), smoking status), as well as comorbidities and the American Society of Anesthesiologists (ASA) classification. Foot and ankle defect-related data included etiology and localization of the defect, with distinctions made between specific locations on the foot, the ankle, and the distal third of the lower leg. The foot and ankle were anatomically defined as beginning at the proximal margin of the ankle joint. The distal third of the lower leg was defined anatomically as extending to the proximal aspect of the malleolus. The peri-operative data set contained the overall operating time, the arterial and venous anastomotic vessels of the recipient site, the anastomosis type (end-to-end or end-to-side) and the flap type. The post-operative data set included information on complications related to the free flap procedure and the necessity for revision surgery.

### 2.2. Controlled Cohort Study Design

All patients who underwent microvascular free flap reconstructions identified were contacted via telephone and were invited to participate in a long-term re-evaluation in our outpatient setting. Patients unable to attend the evaluation in person were provided with study-specific questionnaires via postal delivery. With these patients, the questionnaires were completed together over the phone with a trained member of the study team. Afterward, the completed questionnaires were sent back via postal delivery for further analysis. Patient-reported outcome measures (PROM) were employed to assess long-term functionality, scar quality, aesthetic appearance, health-related quality of life, pain, and footwear. The following PROM were utilized: The Lower Extremity Functional Scale (LEFS), the Patient Scar Assessment Scale (PSAS), the Aesthetic Likert Scale, the Short Form 36 Health Survey Questionnaire (SF-36), and the numeric rating scale for pain (NRS). We recruited a healthy control group (HC) to compare their patient-reported data to the results evaluated in the long-term follow-up. Patients for the control group were recruited by a physician from our study team in the outpatient clinic of our Department of Surgery, Division of Plastic, Aesthetic, and Reconstructive Surgery. These patients were scheduled for minor elective procedures, such as blepharoplasty or mole excision. Only subjects with no history of ankle or foot defects and no history of lower extremity surgery were included as control subjects. To ensure comparability with the free flap group (FFG), we implemented a structured selection process. Before approaching potential controls, we assessed their eligibility based on gender to ensure a comparable distribution of this parameter. Once a preliminary match was identified, we reviewed their medical history to confirm the absence of comorbidities that could affect lower limb function or quality of life. Only after completing this assessment did we invite patients to participate in the study as controls. Due to missing plausibility, we excluded questionnaires assessing scar quality and aesthetic outcome, pain and footwear within the HC.

#### 2.2.1. Patient-Reported Outcome Measures

##### Lower Extremity Functional Scale

The long-term functional outcome following surgery was assessed using the validated German version of the Lower Extremity Functional Scale (LEFS), a highly reliable with 0.98 and internally consistent (Cronbach alpha = 0.95) tool to assess functional impairment across a variety of conditions affecting the lower extremity [25,26]. The LEFS is a patient-reported measure comprising 20 items, with a score ranging from 0 (indicating extreme difficulty or inability to perform activity) to 4 (no difficulty) [25]. By summing the scores of the individual items, the total score can be obtained. Scale scores may range from 0 to 80, with higher scores indicating superior physical functioning. Scores between 61 and 80 are indicative of normal or minimally limited functioning, while scores between 41 and 60 reflect moderate functional limitations. Scores below 40 are classified as severe functional impairments [25,27].

##### Patient Scar Assessment Scale

The scar quality was evaluated using the validated German 2.0 version of the Patient Scar Assessment Scale (PSAS). It is a reliable, widely validated and frequently used scare assessment scale to measure scar quality in all types of scares (reliability = 0.73, internal consistent with Cronbach alpha = 0.76) [28]. In this study, the objective was to exclusively analyze the patients’ subjective view of the foot and ankle without considering the observers’ view. The patient-reported scale comprises six items, each rated on a scale of 1 to 10, and assesses the following characteristics: pain, itch, color, stiffness, thickness, and irregularity. The total score is calculated as the sum of the individual item scores, with higher scores reflecting a poorer quality of scar. Additionally, an item designated as “overall impression” is rated on a 1 to 10 scale. However, this item is not included in the total score and is analyzed independently [28,29].

##### Aesthetic Likert Scale

A 5-point Likert scale, a non-validated subjective questionnaire in German for evaluating aesthetic outcomes of free flap reconstructions, was used to assess patient satisfaction with the aesthetic appearance of the reconstructed limb. The 5-point Likert scale was designed to evaluate four specific attributes pertaining to the reconstructed ankle and foot region: shape, color, texture and general appearance. Each item can be evaluated on a scale of 1 to 5 points. A score between 17 and 20 is indicative of an excellent aesthetic outcome, while a score between 14 and 16 is indicative of a good result. A score of 10 to 13 points is classified as mediocre. An aesthetic result is deemed unsatisfactory when the patient scores between 7 and 9 points. A score between 4 and 6 points indicates that the patient is completely dissatisfied with the aesthetic outcome. The questionnaire has previously been employed in the subjective evaluation of aesthetic outcomes of free flaps in lower extremity reconstruction [17].

##### Short-Form 36 Health Survey Questionnaire

We used the widely validated German version 1.0 of the SF-36 to assess the post-operative health-related quality of life (HRQoL). The HRQoL is concerned with the impact of health on an individual’s capacity to lead a fulfilling life. It represents the extent to which an individual’s daily life is affected by health issues, encompassing physical, emotional, and social factors [27,30,31]. The patient-reported questionnaire comprises 36 items which can be summarized into eight domains: pain, physical functioning, vitality, general health perceptions, physical role functioning, emotional role functioning, social role functioning, and mental health/emotional wellbeing. The scores for each domain were calculated using standard norm-based scoring methods [32,33]. The SF-36 is the most widely used and well-validated health status instrument and one of the most responsive and most extensively tested PROMs with a reliability up to 0.92 and an internal consistency up to 0.83 [34].

##### Numeric Rating Scale for Pain

The subjective pain was evaluated using the 10-point Numeric Rating Scale (NRS), which is considered as the first-choice measure of pain intensity. Patients were required to quantify their pain on a scale from 0 (no pain) to 10 (the worst pain imaginable). We evaluated the patients’ pain both at rest and during activity [35,36].

##### Footwear

A brief, non-validated questionnaire, adapted from a previous study on the long-term outcomes of microsurgical reconstructions of the plantar foot, was devised to evaluate the footwear worn by patients [7]. The objective was to ascertain whether patients wore regular footwear, orthopedic shoes, or orthopedic insoles. Moreover, we investigated whether patients experienced side effects associated with footwear, such as discomfort or irritation from pressure points [37].

### 2.3. Statistical Analysis

All statistical analyses were performed using IBM^®^ SPSS^®^ (Statistics 28, Armonk, North Castle, NY, USA). The statistical evaluation included the calculation of means or medians, as well as the determination of standard deviations (SD) or ranges for continuous or ordered variables. Additionally, relative frequencies were ascertained for categorical factors. In instances where data exhibited a parametric distribution, the Shapiro–Wilk test was employed for analysis. Continuous data were examined using two-tailed t-tests or the non-parametric Mann–Whitney U test, while Fisher’s exact test was applied for categorical data analysis. Bivariate correlations were analyzed using Pearson correlations with the exception of gender, smoke status complications and revisions, which were calculated with Spearman correlations. We performed a sample size calculation using G*Power (Heinrich Heine University Düsseldorf, Düsseldorf, Germany; Version 3.1.9.4). The power analysis indicated a minimum requirement of 12 patients in total to achieve a power of 80% with a significance level of 0.05. This calculation was based on previous studies using the LEFS in patients with lower leg reconstructions [38] as well as in healthy control patients [39]. A *p*-value of less than 0.05 was considered statistically significant.

## 3. Results

### 3.1. Demographic Data

**Figure 1:**
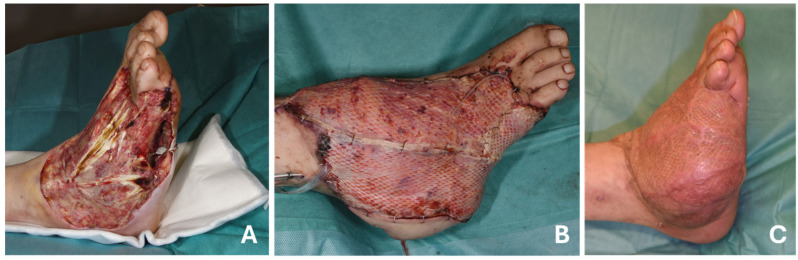
Reconstruction of an extensive foot defect using a free latissimus dorsi flap: (**A**) pre-operative foot defect with exposed functional structures; (**B**) post-operative foot reconstruction with free latissimus dorsi flap in combination with split-thickness skin graft; (**C**) long-term outcome of the recipient site at a 1.5-year follow-up.

Of the 55 potential patients who were eligible for inclusion in the study, only 13 (23.6%) agreed to participate. The cohort consisted of 84.6% male (11/13) and 15.4% female (2/13) participants, with a mean age of 50.9 years (SD ± 11.9) at the time of reconstructive surgery. The mean age at the time of follow-up was 59.0 years (SD ± 10.4). The mean follow-up period was 8.6 years (SD ± 5.2) years. A demographic overview of the patient cohort is given in Table 1. A total of 16 healthy control subjects were included in the study, consisting of 87.5% men (*n* = 14) and 12.5% (*n* = 2) women, with a mean age of 51.1 years (SD ± 11.3). No significant differences were observed in gender (*p* = 0.617), age (*p* = 0.063), BMI (*p* = 0.541) and smoking status (*p* = 0.513) between the FFG and the HC. We found significant differences in every comorbidity between FFG and HC, with the HC being significantly healthier (respectively *p* < 0.001). No significant correlations could be found between gender, age at trauma, BMI, smoke status and complications, revisions and follow-up (Table S1). A defect situation following a soft tissue infection of the right foot is presented, along with post-operative images showing foot reconstruction with a free latissimus dorsi flap and the long-term outcome of the recipient site at a 1.5-year follow-up, as depicted in Figure 1.

### 3.2. Defect Characteristics and Peri-Operative Data

The most common indications for free flap reconstructions within our study cohort were traumatic injury and infection (Table 2). In the overall cohort, the distal third of the lower leg was affected in 38.5% (5/13) of cases. With regard to the foot, the dorsum was affected in 30.8% (4/13) of patients. Table 2 provides a comprehensive overview of the etiologies and locations of all defects. The most utilized free flap was the gracilis free flap. This finding was observed in 53.8% (7/13) of all patients. In total, six different flap types were utilized for reconstructive procedures within the cohort (Table 2). Four patients (30.8%) received external fixation, and two patients (15.4%) underwent open reduction and internal fixation.

### 3.3. Flap-Related Complications and Revision Surgery

Table 3 presents an overview of complications and revision surgeries relating to flaps. The flap success rate within the FFG was 100% (13/13). Flap-specific complications were observed in 38.5% (5/13) of all patients. The most prevalent complications were wound healing disorders (30.8%, 4/13) and infections (15.4%, 2/13). Wound healing disorders were observed in 14.3% (1/7) of gracilis free flaps and 50.0% (1/2) of lateral arm flaps with such disorders being present in every other used flap type. Infection was observed in one case of the latissimus dorsi flap and one case of the scapula flap. Furthermore, one of the two lateral arm flaps exhibited evidence of thrombosis. Revision surgery was performed in four of the 13 patients (30.8%).

### 3.4. Long-Term Follow-Up

#### 3.4.1. Long-Term Functional Outcome

The mean total LEFS score was significantly lower within the FFG (42.5 ± 20.5) compared to the healthy controls (68.7 ± 19.6) (*p* = 0.002), indicating that the patients in our prospective cohort are experiencing moderate functional limitations in day-to-day activities (Table 4). The total LEFS score showed a significant negative correlation with the NRS at rest (*p* = 0.015) and during activity (*p* = 0.041) (Table S2). Additionally, a positive association was found with the total score of the Aesthetic Likert scale (*p* = 0.009) (Table S2). A significant negative association was observed between smoking status and LEFS scores (*p* = 0.005) (Table S3).

#### 3.4.2. Long-Term Health-Related Quality of Life and Pain

When compared to the healthy controls, mean scores were significantly lower within the prospective cohort regarding the subcategories pain (*p* = 0.019), physical functioning (*p* = 0.013), and physical role functioning (*p* = 0.006). No significant differences were observed in vitality, emotional role functioning, social role functioning, mental health, or general health perception (Table 4). Most of the subcategories (physical functioning, physical and emotional role functioning, vitality, pain and general health of SF-36 showed a significant negative correlation between LEFS scores (Table S2). A significant negative association was found between smokers and the SF-36 subcategories of physical and emotional role functioning, emotional well-being, pain, social functioning, and general health (Table S3). The mean NRS score as reported in the FFG was 1.0 (SD ± 1.3) for rest and 3.3 (SD ± 3.2) for activity. Vitality, emotional well-being, and general health were significantly negatively associated with pain at rest and during activity (Table S2). No associations were found between NRS at rest or during activity and demographic or surgery-related characteristics (Tables S3 and S4).

#### 3.4.3. Long-Term Scar Quality and Aesthetic Satisfaction

Using the patient-reported PSAS, a mean total score of 23.5 (SD ± 13.5) was reported. Using the 5-point Aesthetic Likert Scale, patients achieved a mean score of 14.3 (SD ± 4.4), indicating a favorable aesthetic outcome. Table 4 presents the results for all subscales, as well as the overall scores for the patient-reported PSAS and the 5-point Aesthetic Likert Scale. We found significant negative associations between age at follow-up and PSAS sub-scores for pain, color, stiffness, thickness, and irregularity. No significant associations were found for the PSAS sub-score itching (Tables S5 and S6) in relation to demographic and surgery-related characteristics. The total score of the Aesthetic Likert Scale was significantly positively associated with the following subcategories of the SF-36: vitality, emotional well-being, pain, and general health (Table S2). Smoking status had a significant impact on perceived texture in the Aesthetic Likert Scale, whereas no associations were found for the other sub-scores of the Aesthetic Likert Scale concerning demographic and surgery-related characteristics (Tables S5 and S6).

#### 3.4.4. Long-Term Usage of Footwear

The majority of the individuals of the FFG (61.5%, 8/13) stated that they required orthopedic footwear or insoles due to the free flap transfer (Table 4). A total of 53.8% (7/13) of patients reported experiencing footwear-related issues since receiving the free flap with most of them (71.4%, 5/7) already using orthopedic shoes or insoles. Patients undergoing reconstruction of the distal third of the lower leg (n = 5) predominantly wore normal shoes (3/5, 60%), while the majority of patients undergoing reconstruction of the foot/ankle (6/8, 75%) wore orthopedic footwear.

## 4. Discussion

The foot and ankle are integral to the mobility that is a fundamental aspect of human functioning. Trauma to this area can have a profound impact on an individual’s physical and mental well-being affecting their ability to engage in daily activities, work, and social interactions [12,24]. In cases of extensive soft tissue defects, injuries located distally, and exposed bones or tendons, conventional methods such as skin grafts, local flaps, and pedicled flaps may prove inadequate for providing adequate coverage. In order to address these challenges, free flaps have become a pivotal tool for achieving effective defect coverage in complex lower extremity trauma [3,4,5]. The use of free flaps in microsurgical reconstruction for severe lower limb injuries has significantly reduced amputation rates and improved functional outcomes [12,36,37]. Despite relatively high rates of post-operative complications, it remains a safe and reliable approach for addressing intricate defects, with flap success rates exceeding 90% [20,38,39,40,41,42,43].

The aim of this study was to assess the long-term functional outcomes and health-related quality of life in patients who had undergone microvascular reconstructions of the foot and ankle region. The influence of various factors, such as scar quality, aesthetic satisfaction, pain, and footwear use, was evaluated from a patient-centric perspective. To this end, a patient-centric approach was employed, with the use of patient-reported outcome measures (PROMs) to assess the patients’ self-perspective regarding the long-term results. The data presented herein offer valuable insights into patients’ daily activities, social interactions, emotional state and overall well-being [11,44,45]. Although patients exhibited satisfactory long-term functional outcomes, they did not attain the same degree of perceived functionality as the healthy control cohort. Patients reported that they continued to experience limitations in certain aspects of their health-related quality of life. Significant limitations were reported with regard to daily activities and footwear choices. Moreover, they reported significant levels of pain during physical activities. The following sections present our findings regarding long-term functionality, health-related quality of life, aesthetic satisfaction, scar quality, pain, and footwear usage.

### 4.1. Long-Term Functionality After Microsurgical Reconstruction of Foot and Ankle Defects

While the main objective of free frap transfers in foot and ankle defects is limb salvage, it is imperative to restore physical function for the patient’s overall quality of life [12]. Despite the success of the reconstructive procedure from the surgeon’s perspective, patients continue to experience long-term functional limitations, as shown in our controlled study cohort. We evaluated a mean LEFS score of 42.5 (±20.5), indicating a moderate long-term impairment of physical functionality of the lower extremity. This score is significantly lower than that observed in the HC (mean LEFS: 68.7 ± 19.6, *p* = 0.002). These findings are consistent with the current literature, which predominantly reports moderate functional impairments, with mean LEFS scores ranging from 45.1 to 64.7 in studies evaluating long-term results after free flap reconstructions of the lower extremity [12,46,47,48,49]. In cases of severe foot trauma, it can be challenging for both the patient and the surgeon to determine whether to pursue limb salvage or amputation [11,49,50]. Although amputation results in a lower incidence of complications and re-operations and is associated with shorter hospitalization and an earlier return to work, individuals who undergo this procedure are prone to chronic (phantom) pain and a higher prevalence of physical and mental limitations [6,12,51,52]. A study by Krijgh et al. [12] analyzed the long-term outcomes regarding mental health and physical function in patients with severe lower limb trauma. In addition to the moderate physical impairment observed in patients with lower extremity injuries treated with free flaps, those who undergo amputation are more likely to experience higher levels of physical impairment than those who receive free flaps [12].

In light of the unique anatomical and functional demands of the foot, it is noteworthy that patients who underwent microsurgical reconstruction of defects in the weight-bearing plantar foot area exhibited moderate limitations in lower extremity physical functionality [46]. The literature on challenges in the lower extremity is extensive, with evidence indicating that proximal leg injuries tend to result in superior functional outcomes compared to those involving the foot and ankle [37]. The functional outcomes related to the anatomical involvement of the lower extremity were similar in our patient cohort (Table 4). Our results showed strong associations between perceived long-term functionality and pain, aesthetic perception, smoker status as well as the most aspect of HRQoL (Tables S2 and S3). These results indicate an intricate interplay between physical, psychological, and social factors [21]. Individuals with physical disabilities are constrained not only in their daily activities and work life but also in their opportunities for social interactions and meaningful interpersonal exchange. This, in turn, can contribute to social isolation, a decline in mental health, and a reduction in overall quality of life. This underscores the strong interdependence between functional ability and specific domains of quality of life [53].

### 4.2. Long-Term Health-Related Quality of Life After Microsurgical Reconstruction of Foot and Ankle Defects

The term HRQoL describes a multidimensional concept that refers to the impact of an individual’s health status—including physical, mental, emotional, and social well-being—on their overall quality of life. It encompasses the subjective assessment of the effects of disease, disability, or treatment on an individual’s daily functioning and well-being from that individual’s perspective. HRQoL is concerned with the impact of health on various aspects of an individual’s life, including physical functioning, pain, fatigue, emotional distress, and the ability to engage in social roles and activities. It does not encompass broader life circumstances such as financial or environmental factors [30,54]. The interconnectivity between physical limitations, mental health, social relationships, and emotional well-being is profound, with each domain exerting a substantial influence on the others [12,51]. Severe lower limb trauma can result in life-altering physical disabilities that can limit social interactions and contribute to declines in mental health and emotional well-being, ultimately leading to a reduction in HRQoL [13]. In this study, the widely used and validated SF-36 was employed to assess the patients’ long-term HRQoL [32,33].

In accordance with perceived functionality (LEFS), our FFG exhibited significantly diminished scores in the domains long-term “physical functioning” (*p* = 0.013), “physical role functioning” (*p* = 0.006), and “pain” (*p* = 0.019) when compared to the HC (Table 4). These comparative results of the physical component of the SF-36 are consistent with those of the normative German sample for individuals aged 50–59 years old [54], providing further evidence for the comparability of our small healthy control group. In accordance with the long-term functionality outcomes, patients with distal thigh defects exhibited superior scores in physical components of the SF-36 questionnaire compared to those involving the foot and ankle (Table 4) [54]. In a recent study regarding microsurgical reconstruction of the lower extremity, long-term HRQoL was assessed with the SF-36 questionnaire [47]. Despite comparable patient age and shorter follow-up time with a median of 4.0 years, the long-term results for the physical components of SF-36 were found to be superior in the study by List et al. compared to our FFG group [47]. In their cohort (n = 100), 15% of individuals were identified as smokers, whereas in our FFG (n = 13), the prevalence of smoking was 46.2% [47]. Individuals who smoke tend to report lower SF-36 scores across both physical and mental health components [55]. These results align with the findings in our cohort, where a significant association was observed between smoking status and the SF-36 subcategories of physical and emotional role functioning, emotional well-being, pain, social functioning, and general health (Table S3). Furthermore, our cohort included a higher proportion of foot and ankle defects, which are associated with lower physical function compared to more proximal injuries [37].

In contrast to the notable discrepancies observed in the physical components of the SF-36, no statistically significant differences were identified in any of the mental components (vitality, social functioning, emotional role functioning, and emotional well-being) when comparing our FFG to the HC (Table 4). Just as in the physical components in the HC, comparable results were observed in the mental components with the normative German sample for individuals aged 50–59 years old [54]. In light of these findings, it can be concluded that microsurgical reconstruction of lower extremity defects exerts a long-term influence on specific physical components, rather than on mental aspects. A review of the literature reveals that individuals who have undergone amputation due to complex lower extremity trauma are at an elevated risk of developing chronic pain, physical and mental limitations, depression, and a reduced HRQoL when compared to patients who have undergone reconstruction [6,51,52,56]. The necessity for amputation represents a significant and distressing life event for many patients, rendering limb salvage a more psychologically acceptable alternative to amputation [57]. With regard to the emotional aspects of the SF-36, amputation can exert a range of effects. The available evidence suggests that amputees tend to report lower scores in the domains of “emotional well-being” and “emotional role functioning”. Factors such as body image issues, depression, and challenges in fulfilling emotional roles contribute to a reduction in emotional well-being. Furthermore, individuals who have undergone amputation may encounter constraints in their social activities and interactions, which can be attributed to both physical limitations and social stigma associated with the condition [58].

Although the long-term SF-36 scores were relatively favorable, our patients reported long-term limitations in specific physical health aspects of HRQoL. Conversely, no significant long-term impact was observed in the mental health aspects of the SF-36. Nevertheless, mental health is an important determinant of physical health [12]. Although our patients exhibited mental component scores comparable to those of our HC, it is imperative to integrate psychological support, physical therapy, and occupational therapy as integral components of standard care for patients experiencing severe lower limb trauma [7,12,20]. It is, therefore, feasible to facilitate the reintegration of patients with physical or mental impairments following major lower extremity injuries into daily routines, social networks, and work life in the long-term [53,59].

### 4.3. Long-Term Aesthetic Satisfaction and Scar Quality After Microsurgical Reconstruction of Foot and Ankle Defects

Aesthetic satisfaction is closely associated with enhanced HRQoL scores [20]. Patients’ perceptions of their overall well-being are significantly influenced by the appearance of their reconstructed limb, underscoring the importance of considering aesthetic factors as integral components of lower limb reconstruction [19]. This is supported by our data, where we found a strong positive association between aesthetic satisfaction and emotional well-being, as well as other HRQoL components such as vitality, pain, and general health (Table S2). Despite improvements in flap success rates, the majority of patients remain dissatisfied with aesthetic outcomes, particularly regarding bulkiness and loss of contour, which were identified as key aesthetic concerns [7,17]. Additionally, scarring not only visibly impacts the aesthetic outcome but also leads to symptoms such as erythema, burning sensations, pruritus, and pain. Such symptoms can cause considerable discomfort, thereby further reducing HRQoL [60]. This study revealed that long-term outcomes for perceived aesthetic appearance and scar quality were satisfactory to good. The scar quality was reported to be favorable (pain, itch) to moderate (color, stiffness, thickness, and irregularity). In contrast, the aesthetic appearance, including form, color, and texture, was perceived as good. In our FFG, color was identified as the most negatively perceived aesthetic factor. However, it is important to note that these findings are descriptive in nature and should be interpreted with caution. In contrast to physical functioning and HRQoL, no notable discrepancies were observed between patients with foot and ankle trauma and those with distal tibia trauma with respect to aesthetic satisfaction and scar quality.

The importance of the aesthetic outcome for patients is further highlighted by the frequent desire for secondary refinement following lower extremity free flap surgeries [19]. In a study conducted by Nelson et al. [19], secondary aesthetic revision was performed on 21.1% of patients primarily due to concerns regarding bulkiness [19]. However, comparable results were observed in our study, as 23.1% of our FFG underwent secondary aesthetic refinement surgery (Table 3). Bulkiness and loss of contours can contribute to a reduction in aesthetic satisfaction and may also have a functional impact, preventing proper shoe fitting and leading to physical limitations. This illustrates the dual impact that aesthetic considerations can have on both the physical and psychological well-being of patients.

### 4.4. Long-Term Pain After Microsurgical Reconstruction of Foot and Ankle Defects

The objective of microsurgical reconstruction is to restore the functionality of the extremity, while also alleviating the pain caused by the defect. This is especially important, as the experience of pain has been demonstrated to have a substantial influence on patient satisfaction [60]. Pain is a complex phenomenon that can have a profound impact on various aspects of an individual’s life, including daily functioning, occupational performance, social relationships, familial roles, and emotional well-being [61,62,63]. Chronic pain was demonstrated to significantly reduce health-related quality of life (HRQoL), affecting both physical and mental health domains [63]. Moreover, pain is a well-documented risk factor for the development of depression and anxiety disorders. Individuals with comorbid mental health disorders frequently report elevated pain intensity, thereby establishing a vicious cycle of pain and psychological distress [63,64]. Additionally, chronic postsurgical pain is regarded as an independent risk factor for long-dysfunction of the lower limb [44].

The SF-36 revealed significant limitations within the subcategory of pain. These results are comparable to those reported in the current literature regarding microsurgical reconstruction of lower extremity defects [20,64]. In accordance with the findings of the SF-36, a mean NRS score of 3.3 (±3.2) for pain during activity was recorded, indicating a clinically significant level of pain [65]. Consistent with the preceding findings of this study, patients with injuries to the foot and ankle reported higher levels of pain during activity than those with defects of the distal tibia. These results could not be confirmed for pain at rest (see Table 4). However, we found significant associations between pain and several aspects of long-term HRQoL, functionality, aesthetic satisfaction, and scar quality. This highlights pain as an important influencing factor in the long-term outcomes of patients undergoing microsurgical reconstruction of the foot and ankle region (Table S2). These findings underscore the necessity of pain management both at rest and during activity, to facilitate comprehensive rehabilitation in patients who have undergone reconstruction of ankle and foot defects.

### 4.5. Long-Term Usage of Footwear After Microsurgical Reconstruction of Foot and Ankle Defects

The absence of contour and the bulkiness of the transferred flap were demonstrated to affect patients’ aesthetic satisfaction and, furthermore, to influence their choice of footwear. The altered anatomy of the foot, along with the thicker regions resulting from the free flap, may pose challenges in wearing standard footwear, thereby indicating the necessity for custom-made shoes [7,12]. Most of the patients (61.5%) in our FFG reported wearing orthopedic shoes or soles. Footwear-related issues were observed in the majority of FFG (53.8%), with most of them (71.4%) already using orthopedic shoes or insoles (Table 4). The potential for poorly fitting shoes, which may be a consequence of the presence of bulky tissue, to result in the formation of pressure sores or ulcerations represents a significant risk. These findings highlight the importance of utilizing pliable flaps that can adapt to the complex contours of the foot and ankle to mitigate the risk of long-term complications [7,43].

## 5. Limitations

This study has several limitations. It should be noted that the cohort study design is inherently limited in its ability to provide conclusive results (e.g., variations in documentation styles and missing data in the retrieval of clinical data). Of a total of 55 potential patients who had received microvascular free flaps for reconstructions, only 13 (23.6%) agreed to participate in this study. This leaves a bias, as patients who were not satisfied with their (long-term) outcome might have been reluctant to participate in this study. The low response rate is consistent with findings from similar research. Depaoli et al. [66] reported comparable challenges in their study on patients with external fixators on the lower limbs. Like our study cohort, their patients underwent demanding treatment and a prolonged recovery period, which they identified as contributing factors to the high rate of non-respondents. Moreover, with a follow-up period of 8.1 years, a certain proportion of non-responders is inevitable. There is evidence suggesting that non-responders do not necessarily have worse clinical outcomes than responders [67]. However, a selection bias due to a higher response rate among more satisfied patients cannot be ruled out. Due to the relatively limited number of patients and the variety of free flap types, a comprehensive analysis of the differences between flap types was not feasible. Although the age difference of 7.9 years between the FFG and HC groups was not statistically significant, it could still be a potential limitation, as age may influence certain aspects of quality of life as well as comorbidities [54,55]. This study was designed as a comparative cohort study. Consequently, it is not feasible to draw conclusions regarding cause and effect. Furthermore, we only used a patient-centric approach with PROMs to evaluate the patients’ perspective regarding long-term results after microvascular free flaps for reconstructions of defects of the ankle and foot region. Without objective data, our findings must be interpreted within this framework of limitations. Additionally, using PROMs to compare patients’ pre- and post-operative outcomes would be highly valuable, offering important insights into patient-perceived recovery trajectories. Another potential bias is that some patients completed the questionnaires once with telephone support, while others did so on-site. Another limitation of this study is the lack of a comparison between patients undergoing free flap transfer and those undergoing amputation for severe lower limb trauma. Such a comparison would be highly valuable, offering insights into patients’ long-term functional outcomes and HRQoL. It could provide crucial data to guide decision-making in cases of life-altering lower limb defects.

## 6. Conclusions

Patients undergoing free flap reconstructions for ankle and foot defects experience moderate long-term physical limitations and persistent pain during activity. Our patient-centric long-term results indicate that ankle and foot defects have a substantial long-term impact on certain physical domains of health-related quality of life, while mental health appears to be relatively less affected. Additionally, the study reported satisfactory-to-good long-term outcomes in terms of aesthetic satisfaction and scar quality. Restoring functionality after severe lower limb trauma is of paramount importance, particularly given the intricate interplay between physical, psychological, and social factors (21). A substantial body of research has demonstrated a strong correlation between physical disabilities, mental health, and social relationships. This evidence highlights the profound impact physical impairments can have on psychological well-being and social integration. Conversely, psychological and social factors significantly influence the physical recovery process, emphasizing the importance of an integrated approach to rehabilitation (12,53).

## Figures and Tables

**Table 1:** Demographic characteristics and ASA scores.

Characteristics, n (%)	FFG	HC
Number of patients	13	16
Gender		
Male	11 (84.6)	14 (87.5)
Female	2 (15.4)	2 (12.5)
Age at surgery (years), mean (SD)	50.9 (11.9)	
Age at follow-up (years), mean (SD)	59.0 (10.4)	51.1 (11.3)
BMI (kg/m^2^), mean/median (SD/range)	26.9/26.5 (4.0/21.1–33.0)	25.9/25.4 (4.3/20.3–36.3)
Non-smoker	7 (53.8)	8 (50.0)
Smoker	6 (46.2)	4 (25.0)
Follow-Up (years), mean/median (SD/range)	8.6/9.4 (5.2/0.9–17.1)	
Comorbidities, n (%)		
Cardiovascular	7 (53.8)	3 (18.8)
Diabetes mellitus	3 (23.1)	2 (12.5)
Neurological	4 (30.8)	0 (0)
Malignancy	2 (15.4)	0 (0)
Musculoskeletal	1 (7.7)	0 (0)
Peripheral Artery Disease	3 (23.1)	0 (0)
Kidney	1 (7.7)	0 (0)
Rheumatic	1 (7.7)	0 (0)
ASA Score, n (%)		
ASA I	3 (23.1)	
ASA II	5 (38.5)	
ASA III	4 (30.8)	
ASA IV	1 (7.7)	

FFG: free flap group; HC: healthy control group; SD: standard deviation; BMI: body mass index; ASA: American Society of Anaesthesiologists classification.

**Table 2:** Defect characteristics, flap types and peri-operative data of FFG.

Etiology, n (%)	FFG (n = 13)
Trauma	5 (38.5)
Infection	4 (30.8)
Vascular disease	3 (23.1)
Malignancy	1 (7.7)
Localization, n (%)	
Distal third of lower leg	5 (38.5)
Dorsum pedis	4 (30.8)
Planta pedis	3 (23.1)
Calcaneus	3 (23.1)
Medial malleolus	1 (7.7)
Achilles tendon region	1 (7.7)
Lateral malleolus	0 (0.0)
Flap Type, n (%)	
Gracilis flap	7 (53.8)
Lateral arm flap	2 (15.4)
Latissimus dorsi flap	1 (7.7)
Scapula flap	1 (7.7)
Vertical rectus abdominis flap	1 (7.7)
Medial femur condyle flap	1 (7.7)
Operation time (minutes), mean/median (SD/range)	384.5/373.0 (99.2/241–571)
Recipient Vessels, n (%)	
A./V. tibialis posterior	7 (53.8)
A./V. tibialis anterior	4 (30.8)
A./V. dorsalis pedis	1 (7.7)
A./V. poplitea	1 (7.7)
Flap Type and Recipient Artery	
GFF—A./V. tibialis anterior	4 (30.8)
GFF—A./V. tibialis posterior	3 (23.1)
LAF—A./V. tibialis posterior	2 (15.4)
LDM—A./V. tibialis posterior	1 (7.7)
SFF—A./V. tibialis posterior	1 (7.7)
VRAM—A./V. poplitea	1 (7.7)
MFC—A./V. dorsalis pedis	1 (7.7)
Arterial Anastomosis, n (%)	
End-to-Side	8 (61.5)
End-to-End	4 (30.8)
Unknown	1 (7.7)

FFG: free flap group; SD: standard deviation; A: Arteria; V: Vena; GFF: gracilis free flap; LAF: lateral arm flap; LDM: latissimus dorsi flap; SFF: scapula flap; VRAM: vertical rectus abdominis flap; MFC: medial femur condyle flap.

**Table 3:** Flap-related complications and revision surgery.

Flap Success Rate, n (%)	13 (100%)
Partial Flap Loss	0 (0.0)
Complete Flap Loss	0 (0.0)
Flap-Related Complications, n (%)	5 (38.5)
Wound healing disorder	4 (30.8)
Haematoma	0 (0.0)
Thrombosis	1 (7.7)
Infection	2 (15.4)
Seroma	0 (0.0)
Flap-Specific Complications Dependent on Location	
Distal third of the lower leg	2
Calcaneus	2
Dorsum pedis	1
Planta pedis	1
Achilles tendon region	1
Revision Surgery, n (%)	4 (30.8)
Number of Revision Surgeries	
1	2 (15.4)
2	1 (7.7)
3	0
4	1 (7.7)
Refinement Surgery, n (%)	
Yes	3 (23.1)
No	10 (76.9)

**Table 4:** Long-term results of functionality, health-related quality of life, scar quality, aesthetic outcome and pain after microvascular free flap reconstruction of the foot and ankle.

	FFG	HC	*p*-Value (FFG vs. HC)	Effect Size	Foot/Ankle	Distal Third of Lower Leg
Number of patients (n)	13	16			8	5
LEFS, mean/median (SD/range) [0–80]	42.5/43.0 (20.5/5–80)	68.7/78.0 (19.6/23–80)	0.002	1.27	37.5/39.5 (19.2/5–68)	50.6/43.0 (21.9/23–80)
SF-36, mean/median (SD/range) [0–100]						
Pain ^b^	55.8/55.0 (34.5/0.0–100)	83.8/100 (27.2/22.5–100)	0.019	0.905	51.3/55.0 (35.1/0.0–100)	63.0/57.5 (36.0/22.5–100)
Physical functioning ^a^	55.0/45.0 (29.7/0.0–100)	83.8/97.5 (27.2/25.0–100)	0.013	0.987	50.0/55.0 (29.3/0.0–80.0)	63.0/45.0 (31.7/35.0–100)
Vitality ^b^	56.5/60.0 (20.6/5.0–80.0)	63.1/67.5 (22.5/25.0–100)	0.422	0.296	53.8/60.0 (22.3/5.0–80.0)	61.0/65.0 (18.8/30.0–80.0)
Physical role functioning ^b^	38.5/25.0 (44.0/0.0–100)	82.8/100 (29.9/0.0–100)	0.006	1.17	31.3/12.5 (43.8/0.0–100)	50.0/25.0 (46.8/0.0–100)
Emotional role functioning ^a^	66.7/100 (47.1/0.0–100)	77.1/100 (37.9/0.0–100)	0.619	0.24	62.5/100 (51.8/0.0–100)	73.3/100 (43.5/0.0–100)
Social functioning ^a^	85.6/87.5 (17.6/50.0–100)	89.1/100 (20.3/50.0–100)	0.374	0.177	82.8/87.5 (18.8/50.0–100)	90.0/100 (16.3/62.5–100)
Emotional well-being ^a^	70.6/80.0 (23.7/28.0–96.0)	78.3/82.0 (18.5/44.0–100)	0.329	0.354	69.3/80.0 (24.3/30.0–96.0)	72.8/80.0 (25.5/28.0–92.0)
General health ^b^	60.4/65.0 (25.8/20.0–90.0)	67.8/65.0 (22.9/30.0–100)	0.418	0.298	58.1/62.5 (25.1/20.0–90.0)	64.0/75.0 (29.5/30.0–90.0)
PSAS mean/median (SD/range)						
Total score [6–60]	23.5/25.5 (13.5/6–46)				22.7/23.0 (13.0/6–38)	24.6/28.0 (15.6/6–46)
Pain	2.2/1.0 (2.0/1–8)				1.6/1.0 (1.0/1–3)	3.0/2.0 (2.9/1–8)
Itch	2.3/1.5 (1.8/1–7)				1.7/1.0 (1.0/1–3)	3.0/2.0 (2.6/1–7)
Color	4.9/5.0 (2.9/1–10)				4.7/5.0 (2.6/1–9)	5.2/5.0 (3.5/1–10)
Stiffness	4.7/5.0 (2.9/1–9)				5.0/6.0 (3.4/1–9)	4.2/5.0 (2.3/1–7)
Thickness	4.5/3.5 (3.0/1–9)				4.6/3.0 (3.5/1–9)	4.4/4.0 (2.6/1–7)
Irregularity	5.0/5.0 (3.5/1–10)				5.1/6.0 (3.6/1–10)	4.8/4.0 (3.7/1–10)
Overall impression [1–10]	4.4/4.0 (3.0/1–9)				4.6/4.0 (3.3/1–9)	4.2/4.0 (2.8/1–7)
Aesthetic Likert Scale, mean/median (SD/range) [4–20]						
Total score	14.3/15 (4.4/5–20)				14.6/16.0 (4.8/5–20)	13.8/13.0 (4.3/9–20)
Form	3.8/4.0 (1.2/1–5)				3.9/4.0 (1.3/1–5)	3.6/4.0 (1.1/2–5)
Color	3.3/3.5 (1.4/1–5)				3.6/4.0 (1.4/1–5)	3.0/2.0 (1.4/2–5)
Texture	3.7/4.0 (0.9/2–5)				3.6/4.0 (1.0/2–5)	3.8/4.0 (0.8/3–5)
General appearance	3.5/4.0 (1.2/1–5)				3.6/4.0 (1.3/1–5)	3.4/3.0 (1.1/2–5)
Pain, mean/median (SD/range) [0–10]						
At Rest	1.0/0.0 (1.3/0–4)				1.2/0.5 (1.6/0–4)	0.8/0.0 (1.1/0–2)
At Activity	3.3/3.0 (3.2/0–9)				4.0/3.5 (3.8/0–9)	2.4/3.0 (2.3/0–5)
Footwear, n (%)						
Regular footwear	5 (38.5)				2 (25.0)	3 (60.0)
Orthopedic shoes	7 (53.8)				6 (75.0)	1 (20.0)
Orthopedic insoles	1 (7.7)				0	1 (20.0)
Problems caused by footwear	7 (53.8)				5 (62.5)	2 (40.0)
Regular footwear	2				1	1
Orthopedic shoes	4				4	0
Orthopedic insoles	1				0	1

FFG: free flap group; HC: healthy control group; SD: standard deviation; LEFS: lower extremity functional scale; SF-36: short-form 36 healthy survey questionnaire; PSAS: patient scar assessment scale.

## Data Availability

The original contributions presented in this study are included in the article/supplementary material. Further inquiries can be directed to the corresponding author(s).

## Supplementary Materials

The following supporting information can be downloaded at: https://www.mdpi.com/article/10.3390/life15050775/s1, Table S1: Correlation analysis of demographic and surgical-related characteristics; Table S2: Correlation analysis of PROMs scores; Table S3: Correlation analysis of demographic characteristics and SF-36, NRS, LEFS; Table S4: Correlation analysis of surgery-related characteristics and SF-36, NRS, LEFS; Table S5; Correlation analysis of demographic characteristics with PSAS and the Aesthetic Likert scale; Table S6: Correlation analysis of surgery-related characteristics with PSAS and the Aesthetic Likert scale.
