# Supplementary material for: Long-Term Functional Outcomes and Quality of Life After Microvascular Reconstruction of Ankle and Foot Defects: A Monocentric Controlled Cohort Study
Source: Life (Basel). 2025 May 13;15(5):775. doi: 10.3390/life15050775 (PMC12113567; doi:10.3390/life15050775)
Supplement: Supplementary file 1 [file life-15-00775-s001.zip › life-3507289-supplementary.pdf]

Supplementary

Long-Term Functional Outcomes and Qualify of Life after Microvascular Reconstruction of Ankle and Foot Defects

Supplementary S1: Correlation analysis of demographic and surgical-related characteristics

	Age-at Surgery	Age-of Defect	Surgery-Time	Complications	Revision	Revision Amount
[a] Sex	-0.627	-0.285	0.342	-0.101	0.284	*NA
p-value	**0.022**	0.345	0.253	0.742	0.347	
[b] Age-at Follow-up	0.899	0.172	-0.174	0.380	0.134	-0.537
p-value	**< 0.001**	0.575	0.569	0.200	0.663	0.463
[b] Follow-Up in years	-0.514	-0.327	-0.338	0.148	0.201	0.653
p-value	0.072	0.275	0.258	0.692	0.511	0.347
[b] BMI	0.221	0.155	0.364	0.127	-0.045	-0.211
p-value	0.469	0.614	0.222	0.680	0.885	0.789
[a] Smoker	-0.371	-0.082	0.000	-0.098	0.051	0.943
p-value	0.212	0.789	1.000	0.751	0.867	0.057
[b] Age-at Surgery	1.000	0.292	0.000	0.254	0.045	-0.928
p-value		0.334	1.000	0.403	0.885	0.072
[b] Age-of Defect	0.292	1.000	0.214	0.380	0.223	-0.406
p-value	0.334		0.482	0.200	0.465	0.594
[b] Surgery-Time	0.000	0.214	1.000	0.042	0.223	-0.802
p-value	1.000	0.482		0.891	0.465	0.198
[a] Complications	0.254	0.380	0.042	1.000	0.843	NA
p-value	0.403	0.200	0.891		**< 0.001**	
[a] Revisions	0.045	0.223	0.223	0.843	1.000	NA
p-value	0.885	0.465	0.465	**< 0.001**		
[b] Amount of Revisions	-0.928	-0.406	-0.802	NA	NA	1.000
p-value	0.072	0.594	0.198			

a= Spearman Correlation (ρ); b= Pearson Correlation (r); NA= Not applicable;

Supplementary S2: Correlation analysis of PROMs scores

	NRS at Rest	NRS at Activity	LEFS Total Score	Aesthetic Likert Scale Total Score	PSAS Total score
**SF-36**					
[b] Physical Functioning	-0.515	-0.384	0.828	0.526	-0.400
p-value	0.105	0.243	**<0.001**	0.079	0.198
[b] Physical Role Functioning	-0.468	-0.391	0.829	0.430	-0.237
p-value	0.147	0.234	**<0.001**	0.163	0.458
[b] Emotional Role Functioning	-0.557	-0.563	0.778	0.574	-0.427
p-value	0.075	0.072	**0.002**	0.051	0.166
[b] Vitality	-0.869	-0.865	0.581	0.641	-0.438
p-value	**<0.001**	**<0.001**	**0.037**	**0.025**	0.155
[b] Emotional Well-Being	-0.741	-0.822	0.541	0.615	-0.383
p-value	**0.009**	**0.002**	0.056	**0.033**	0.219
[b] Social Functioning	-0.355	-0.435	0.516	0.344	-0.212
p-value	0.284	0.181	0.071	0.274	0.508
[b] Pain	-0.769	-0.893	0.787	0.666	-0.735
p-value	**0.006**	**<0.001**	**<0.001**	**0.018**	**0.006**
[b] General Health	-0.846	-0.825	0.723	0.791	-0.381
p-value	**0.001**	**0.002**	**0.005**	**0.002**	0.222
**NRS**					
[b] Pain at Rest	1	0.886	-0.706	-0.844	0.496
p-value		**<0.001**	**0.015**	**<0.001**	0.121
[b] Pain at Activity	0.886	1	-0.621	-0.683	0.646
p-value	**<0.001**		**0.041**	**0.021**	**0.032**
**LEFS**					
[b] Total Score	-0.706	-0.621	1	0.712	-0.481
p-value	**0.015**	**0.041**		**0.009**	0.144
**Aesthetic Likert Scale**					
[b] Total Score	-0.844	-0.683	0.712	1	-0.502
p-value	**<0.001**	**0.021**	**0.009**		0.115
**PSAS**					
[b] Total Score	0.496	0.646	-0.481	-0.502	1
p-value	0.121	**0.032**	0.114	0.115	

b= Pearson Correlation (r); PROM=Patient Reported Outcome Measures; SF-36= Short Form Health Survey-36; NRS= Numeric Rating Scale; LEFS= Lower Extremity Functional Scale, PSAS= Patient Scar Assessment Scale

Supplementary S3: Correlation analysis of demographic characteristics and SF-36, NRS, LEFS

	Sex	Age-Follow-up in years	Follow-up in years	BMI	Smoker
**SF-36**					
Physical Functioning	-0.029	0.166	0.411	0.426	-0.415
p-value	0.926	0.589	0.163	0.147	0.159
Physical Role Functioning	-0.030	0.032	0.157	0.506	-0.656
p-value	0.922	0.918	0.609	0.078	**0.015**
Emotional Role Functioning	-0.331	0.183	-0.054	0.209	-0.838
p-value	0.270	0.550	0.860	0.493	**< 0.001**
Vitality	-0.433	0.199	-0.191	0.055	-0.355
p-value	0.140	0.515	0.531	0.858	0.234
Emotional well-being	-0.631	0.160	-0.380	-0.029	-0.644
p-value	**0.021**	0.602	0.200	0.924	**0.018**
Social functioning	-0.423	0.043	-0.113	0.034	-0.678
p-value	0.150	0.890	0.712	0.912	**0.011**
Pain	-0.577	0.532	-0.135	0.139	-0.835
p-value	**0.039**	0.061	0.660	0.650	**< 0.001**
General health	-0.459	0.074	-0.140	0.113	-0.602
p-value	0.115	0.809	0.647	0.712	**0.029**
**NRS**					
Pain at Rest	0.411	-0.142	0.045	-0.118	0.382
p-value	0.209	0.678	0.895	0.729	0.246
Pain at Activity	0.537	-0.382	0.180	0.076	0.475
p-value	0.089	0.247	0.597	0.824	0.140
**LEFS**					
Total Score	-0.342	0.195	0.226	0.394	-0.723
p-value	0.252	0.523	0.459	0.182	**0.005**

a= Spearman Correlation (ρ); b= Pearson Correlation (r); SF-36= Short Form Health Survey-36; NRS= Numeric Rating Scale; LEFS= Lower Extremity Functional Scale

Supplementary S4: Correlation analysis of surgery-related characteristics and SF-36, NRS, LEFS

	Age at-Surgery	Age-of Defect	Surgery-Time	Complications	Revision	Amount of Revisions
**SF-36**						
Physical Functioning	-0.038	-0.002	-0.198	-0.021	-0.246	-0.289
p-value	0.902	0.994	0.518	0.945	0.417	0.711
Physical Role Functioning	-0.042	0.099	-0.038	0.067	-0.142	-0.623
p-value	0.892	0.749	0.903	0.827	0.644	0.377
Emotional Role Functioning	0.182	-0.172	0.174	-0.074	-0.207	-0.816
p-value	0.551	0.575	0.570	0.811	0.498	0.184
Vitality	0.256	0.245	-0.218	-0.278	-0.541	-0.499
p-value	0.399	0.420	0.475	0.358	0.056	0.501
Emotional Well-Being	0.305	0.030	-0.123	-0.298	-0.538	-0.753
p-value	0.311	0.923	0.690	0.323	0.058	0.247
Social Functioning	0.087	-0.280	0.040	0.022	-0.142	-0.937
p-value	0.778	0.353	0.897	0.942	0.644	0.063
Pain	0.518	0.173	-0.194	0.021	-0.203	-0.641
p-value	0.070	0.572	0.526	0.945	0.506	0.359
General Health	0.126	0.077	-0.341	-0.234	-0.471	-0.692
p-value	0.682	0.804	0.255	0.442	0.104	0.308
**NRS**						
Pain at Rest	-0.141	-0.377	0.227	0.198	0.391	0.327
p-value	0.678	0.253	0.501	0.560	0.234	0.788
Pain at Activity	-0.404	-0.337	0.372	0.246	0.498	0.143
p-value	0.217	0.311	0.260	0.466	0.119	0.909
**LEFS**						
Total	0.070	0.025	-0.050	-0.127	-0.312	-0.506
p-value	0.821	0.934	0.871	0.679	0.299	0.494

a= Spearman Correlation (ρ); b= Pearson Correlation (r); SF-36= Short Form Health Survey-36; NRS= Numeric Rating Scale; LEFS= Lower Extremity Functional Scale

Supplementary S5: Correlation analysis of demographic characteristics with PSAS and the Aesthetic Likert scale

	Sex	Age-Follow-Up in years	Follow-up in years	BMI	Smoker
**PSAS**					
Pain	0.364	-0.758	0.158	-0.034	0.083
p-value	0.244	**0.004**	0.623	0.917	0.799
Itching	0.417	-0.379	0.403	0.135	0.367
p-value	0.178	0.225	0.194	0.676	0.240
Colour	0.328	-0.653	-0.218	-0.186	0.694
p-value	0.298	**0.021**	0.496	0.563	**0.012**
Stiffness	0.427	-0.723	-0.250	0.014	0.621
p-value	0.166	**0.008**	0.433	0.965	**0.031**
Thickness	0.396	-0.694	-0.222	0.163	0.424
p-value	0.203	**0.012**	0.489	0.614	0.170
Irregularity	0.458	-0.834	-0.277	-0.019	0.495
p-value	0.134	**< 0.001**	0.384	0.954	0.102
Overall Opinion	0.493	-0.718	-0.183	0.154	0.348
p-value	0.104	**0.009**	0.569	0.634	0.268
Total Scores	0.391	-0.830	-0.143	0.008	0.541
p-value	0.209	**< 0.001**	0.658	0.981	0.069
**Aesthetic Likert Scale**					
Form	-0.314	0.018	0.034	0.165	-0.468
p-value	0.321	0.956	0.916	0.607	0.125
Colour	-0.066	-0.095	0.077	0.040	-0.395
p-value	0.838	0.768	0.813	0.903	0.203
Texture	-0.446	0.169	0.107	0.077	-0.588
p-value	0.146	0.600	0.741	0.812	**0.044**
Overall Opinion	-0.170	0.008	0.064	-0.073	-0.303
p-value	0.598	0.981	0.844	0.821	0.338
Total Score	-0.196	0.011	0.072	0.054	-0.462
p-value	0.542	0.972	0.825	0.868	0.131

a= Spearman Correlation (ρ); b= Pearson Correlation (r); NA= Not applicable; BMI=Body Mass Index; PSAS= Patient Scar Assessment Scale

Supplementary S6: Correlation analysis of surgery-related characteristics with PSAS and the Aesthetic Likert scale

	Age-at Surgery	Age-of Defect	Surgery-Time	Complications	Revision	Amount of Revisions
**PSAS**						
Pain	-0.728	-0.088	0.218	-0.028	0.115	0.408
p-value	**0.007**	0.785	0.497	0.932	0.721	0.592
Itching	-0.504	-0.078	-0.033	-0.026	0.137	0.833
p-value	0.094	0.810	0.919	0.935	0.671	0.167
Colour	-0.474	-0.060	0.356	-0.273	-0.156	0.414
p-value	0.120	0.854	0.255	0.391	0.629	0.586
Stiffness	-0.522	-0.46	0.207	-0.174	-0.078	0.414
p-value	0.082	0.888	0.519	0.589	0.810	0.586
Thickness	-0.509	-0.254	0.400	-0.100	-0.026	0.277
p-value	0.091	0.426	0.197	0.758	0.936	0.723
Irregularity	-0.607	-0.098	0.343	-0.272	-0.155	0.277
p-value	**0.036**	0.762	0.275	0.392	0.630	0.723
Overall opinion	-0.546	-0.270	0.492	0.000	0.130	0.158
p-value	**0.066**	0.396	0.104	1.000	0.688	0.842
Total scores	-0.661	-0.128	0.327	-0.197	-0.077	0.551
p-value	**0.019**	0.692	0.300	0.540	0.811	0.449
**Aesthetic Likert Scale**						
Form	0.000	0.043	-0.403	-0.496	-0.540	-0.143
p-value	0.999	0.893	0.194	0.101	0.070	0.909
Colour	-0.114	0.100	-0.024	-0.419	-0.257	-0.577
p-value	0.724	0.757	0.940	0.175	0.420	0.609
Texture	0.097	-0.132	-0.282	-0.298	-0.413	-0.327
p-value	0.765	0.682	0.375	0.346	0.182	0.788
Overall Opinion	-0.022	0.128	-0.366	-0.482	-0.379	-0.143
p-value	0.947	0.692	0.242	0.112	0.224	0.909
Total Score	-0.022	0.050	-0.272	-0.413	-0.337	-0.327
p-value	0.946	0.877	0.392	0.183	0.284	0.788

a= Spearman Correlation (ρ); b= Pearson Correlation (r); PSAS= Patient Scar Assessment Scale
